# Approaching the alloy limit of thermal conductivity in single-crystalline Si-based thermoelectric nanocomposites: A molecular dynamics investigation

**DOI:** 10.1038/srep09579

**Published:** 2015-04-08

**Authors:** Ruiqiang Guo, Baoling Huang

**Affiliations:** 1 Department of Mechanical and Aerospace Engineering, The Hong Kong University of Science and Technology, Clear Water Bay, Kowloon, Hong Kong; 2 The Hong Kong University of Science and Technology Shenzhen Research Institute, Shenzhen. 518057, China

## Abstract

Single-crystalline Si-based nanocomposites have become promising candidates for thermoelectric applications due to their prominent merits. Reducing the thermal conductivity *κ* without deteriorating the electrical properties is the key to improve their performance. Through non-equilibrium molecular dynamics simulations, we show that *κ* of single-crystalline Si-based nanocomposites can be reduced to the alloy limit by embedding various nanoinclusions of similar lattice constants but different lattice orientations or space symmetries with respect to the matrix. The surprisingly low *κ* is mainly due to the large acoustic phonon density of states mismatch caused by the destruction of lattice periodicity at the interfaces between the nanoinclusions and matrix, which leads to the substantial reduction of phonon group velocity and relaxation time, as well as the enhancement of phonon localization. The resulting *κ* is also temperature-insensitive due to the dominance of boundary scattering. The increase in thermal resistance induced by lattice structure mismatch mainly comes from the nanoinclusions and the channels between them and is caused by the enhanced boundary scattering at the interfaces parallel to the heat flux. Approaching the alloy limit of *κ* with potentially improved electrical properties by fillers will remarkably improve *ZT* of single-crystalline Si-based nanocomposites and extend their application.

In recent years, bulk semiconductor nanocomposites, i.e., bulk semiconductors with nanoinclusions, have become promising candidates for large-scale thermoelectric applications due to their prominent performance, low cost and structural stability^1^,^2^,^3^,^4^,^5^. The efficiency of thermoelectric materials is often measured by a dimensionless figure of merit *ZT* = *S*^*2*^*σT*/(*κ_L_* + *κ_e_*), where *S* is the Seebeck coefficient, *σ* is the electrical conductivity, *T* is the temperature, and *κ_L_* and *κ_e_* are the lattice and electronic contributions to the thermal conductivity *κ*, respectively. With the nano-size inclusions of a characteristic dimension comparable to the phonon mean free path (MFP) or wavelength, nanocomposites can strongly suppress the phonon transport with less deterioration in electrical properties through classical and quantum confinement effects, therefore leading to a much enhanced *ZT*. Remarkable *ZT* values or improvements over the bulk materials have been reported for AgPb_m_SbTe_2+m_ systems^1^ (*ZT* = 2.2 at 400K), In_0.53_Ga_0.47_As/ErAs nanocomposites^2^ (200% improvement), p-type BiSbTe nanocomposites^3^ (*ZT* = 1.4 at 373K) and PbTeSrTe systems^5^ (*ZT* = ~2.2 at 915K), which are comparable with those of low-dimensional systems^6^,^7^,^8^,^9^,^10^,^11^. More importantly, the nano-bulk combination provides an opportunity to engineer some materials that are unsuitable for thermoelectric applications in their bulk form, such as single-crystalline Si (*ZT* ≈ 0.01 at 300K due to its high *κ_L_* = 148w/mK^12^), as low-cost high-performance thermoelectrics. For single-crystalline Si-based thermoelectric materials, a crystalline matrix is preferred to achieve high charge mobility. However, *κ* of a single crystal is generally much higher than that of its alloy, which is widely believed to be the lowest attainable *κ* in crystalline solids (alloy limit)^2^. Nanopores have been recently introduced to reduce *κ* of Si, leading to significantly enhanced *ZT*. Tang et al.^13^ reported a *ZT* = 0.4 at 300K for nanopatterned holy Si films by decreasing *κ* to ~2W/mK. Yu et al.^14^ and Hopkins et al.^15^ also found that *κ* of Si nanomeshes could be as low as 1.9W/mK and 6.8W/mK respectively without compromise on electrical properties at room temperature. Very recently, Yang et al.^16^,^17^ predicted a *ZT* as high as 0.76 at 300K for n-type three-dimensional Si phononic crystals, in which *κ* can be reduced by a factor up to 500 with little deterioration in the electrical properties compared with those of bulk Si.

With a similar structure, Si-based nanocomposites have rarely been experimentally investigated for thermoelectric applications. The open question is whether *κ* of Si-based nanocomposites can be further reduced so as to approach the alloy limit. The key to answer this lies in a fundamental understanding of thermal transport in nanocomposites. A few simulation works have been conducted to investigate *κ* of nanocomposites using Boltzmann transport equation (BTE)^18^, Monte Carlo (MC)^19^ and molecular dynamics (MD) simulations^20^. These investigations showed that *κ* depends on both the volumetric fraction and size of the nanoinclusions and proposed to reduce *κ* using a high interface density. Some theoretical models have also been developed for the quantitative prediction of the effects of nanoinclusions on *κ*. Kim et al.^21^ proposed an approximate analytical solution to estimate the phonon scattering cross section of polydispersed spherical nanoparticles. Mingo et al.^22^ calculated *κ* of the nanoparticle-in-alloy nanocomposites and predicted a potential 5-fold increase in *ZT* at room temperature. All these investigations demonstrate the great potential of decreasing *κ* of Si-based nanocomposites, which is generally attributed to the enhanced interfacial scattering. However, further efforts are still needed to explore the microscopic origin of the strong interfacial scattering and the strategy to approach the alloy limit in Si-based nanocomposites. It is of importance to illuminate how the various dissimilarities between the nanoinclusions and matrix as well as the design parameters influence *κ* of Si-based nanocomposites and the phonon transport properties such as group velocity and relaxation time. These phonon transport details are highly desired for understanding the *κ* reduction mechanism and are helpful for the design of nanocomposites.

In this work, we investigate *κ* of single-crystalline Si-based nanocomposites using nonequilibrium molecular dynamics (NEMD) simulations and conduct a systematic phonon transport analysis in both frequency domain and real space domain. Various nanoinclusions of similar lattice constants but different mass densities, lattice orientations or space symmetries from those of the matrix are considered. *κ* of samples with various nanoinclusion sizes and pitch spacings are calculated. The influence of different nanoinclusions on *κ* of the nanocomposites is revealed. We find that lattice orientation or space symmetry mismatches can even reduce *κ* of nanocomposites to the alloy limit by depressing the group velocity and relaxation time, as well as enhancing phonon localization. To further the understanding of *κ* reduction in these nanocomposites, the local real-space thermal transport and resistance distribution in the Ge/Si nanocomposites are illuminated and the correlation between the enhanced scattering and the surface vibration states is discussed.

## Results

**Figure 1:**
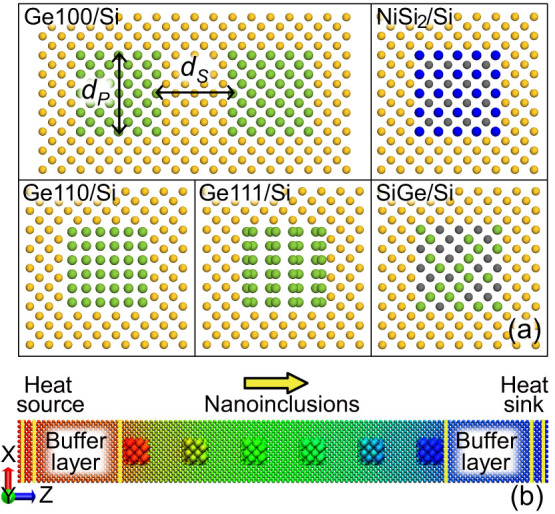
(a) Representative configuration of Si-based nanocomposites characterized by the structural parameters: nanoinclusion size (*d_P_*) and spacing (*d_S_*). Typical unit cells of nanocomposites embedded with Ge100 (Ge nanoinclusions with the crystal orientation [100] parallel to the *Z* axis), Ge110, Ge111, Si_0.5_Ge_0.5_ alloy and NiSi_2_ nanocrystal are also shown. Each ball represents an atom, colored by yellow for Si in the matrix, green for Ge, blue for Ni and gray for Si in the nanoinclusions. (b) Schematic of NEMD simulation domain for the direct method. The heat flux is applied along the *Z* direction and passes through the nanoinclusions in the intermediate region, which is separated from the heat source/sink by a buffer layer to eliminate the nonlinear effects.

The representative configurations of Si-based nanocomposites are shown in Fig. 1(a). The cylindrical nanoinclusions with a square cross section *d_P_* *d_P_* are embedded in the matrix and separated by the spacing *d_S_*. The nanocomposites are constructed by embedding nanoinclusions into bulk Si of 100 orientation (parallel to the *Z* axis) at the designed positions. Eight types of nanoinclusions are considered, including Ge100 (Ge nanocrystal with orientation 100 parallel to the *Z* axis), Ge110, Ge111, Si110, Si111, Si_0.5_Ge_0.5_ alloy (Fd3_m group), NiSi_2_ nanocrystal (Fm3_m group) and pores. Ge, SiGe and NiSi_2_ are good candidates because their lattice constants are very close to that of Si and the electron transport is expected to be little affected.

We calculated *κ* using NEMD with a typical simulation domain shown in Fig. 1(b) and the modified embedded atom method (MEAM) potentials^23^, which have been successfully applied to describe a wide range of materials, in particular semiconductors such as Si, Ge and their alloys^24^,^25^,^26^,^27^. It is noted that for Si and Ge the MEAM potentials can well reproduce the acoustic phonon transport properties but overestimate the frequencies of optical phonons, which can be addressed by 2NN MEAM potentials^28^,^29^. Considering that optical phonons contribute little (~5% for bulk Si) to the thermal conductivity^30^,^31^, the MEAM potentials can still lead to reasonable accuracy for these materials. We first verified the accuracy of these potentials by calculating the bulk *κ* of various samples at 300K. The NEMD simulations give 145 20W/mK for Si, 71 11W/mK for Ge, 4.9 0.6W/mK for Si_0.5_Ge_0.5_ and 7.3 0.7W/mK for NiSi_2_, respectively. These values are in good agreement with the experimental results (148W/mK for Si^12^, 59.9W/mK for Ge^12^, 4.6W/mK for Si_0.5_Ge_0.5_^32^ and ~10W/mK for NiSi_2_^33^), indicating the suitability of these potentials for the targeted investigations.

**Figure 2:**
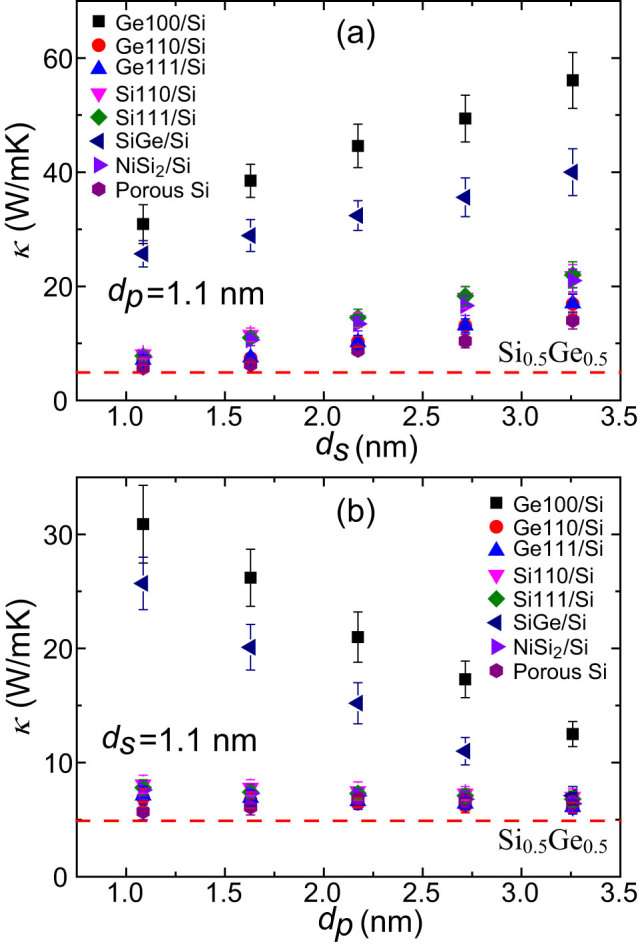
Thermal conductivities of Si-based nanocomposites in the direction perpendicular to the nanoinclusions at 300K, as a function of *d_S_* (fixed *d_P_* = 1.1nm) (a) and *d_P_* (fixed *d_S_* = 1.1nm) (b), in comparison with that of bulk Si_0.5_Ge_0.5_.

Figures 2(a) and (b) show *κ* of all samples with infinite length in the direction perpendicular to nanoinclusions at 300K, as a function of *d_S_* and *d_P_*, respectively, in comparison with that of bulk Si_0.5_Ge_0.5_. For nanoporous structures, to evaluate their applicability as thermoelectric materials, *κ_eff_* based on the effective cross section should be used due to the existence of pores, which is related to the porosity *ϕ* and commonly used *κ_A_* based on the total cross section though 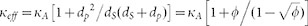
34. A similar treatment has also been employed by Hopkins et al.^15^. It is shown that *κ* of all nanocomposites are significantly reduced with respect to that of bulk Si and the reduction depends on both the configuration and the type of nanoinclusions. Figure 2(a) shows that *κ* of the samples increase almost linearly with the increasing *d_S_*, indicating that important phonon modes are in the ballistic transport regime within this *d_S_* range. This is also supported by the investigations of spectral contributions to *κ*^30^,^31^,^35^,^36^, which found that phonon modes with MFPs from tens of nanometers to a few micrometers contribute significantly to *κ* of bulk Si while those with MFPs < 10nm have negligible contributions. Compared with other samples, *κ* of Ge100/Si and SiGe/Si grow much faster with the increasing *d_S_*. Figure 2(b) shows that *κ* of Ge100/Si and SiGe/Si decrease significantly with the increase of *d_P_* while those of other samples almost remain constant. The comparison between Figs. 2(a) and 2(b) indicates that *κ* is more sensitive to *d_S_*. For the sample Ge100/Si, *κ* decreases by 33.5W/mK with an increase of the volumetric fraction of Ge by 18.8% when *d_S_* decreases; however, by varying *d_P_* *κ* only decreases by 16.4W/mK although the volumetric fraction of Ge increases by 31.3%. The later analysis will show that this is due to the strong scattering at the interfaces parallel to the heat flux. Because the intrinsic MFPs of important phonon modes in the matrix are much longer than those of nanoinclusions, the conductance of channel regions will be more strongly affected by *d_S_* compared with the influence of *d_p_* on that of nanoinclusions and thus leads to more significant variation of *κ* in the nanocomposites.

For fixed configurations, nanoporous Si exhibits the lowest *κ* for most cases; however, *κ* of some nanocomposites can be as low as that of nanoporous Si. Considering that some nanoinclusions can even improve electrical properties, Si-based nanocomposites might achieve a better thermoelectric performance than nanoporous Si. Furthermore, appropriate patterning can even make *κ* of Si-based nanocomposites approach that of bulk Si_0.5_Ge_0.5_ alloy, which probably results in an optimal *ZT*. Also, the type of nanoinclusions significantly influences *κ*. The thermal conductivity of Ge100/Si is larger than that of SiGe/Si, which is reasonable when considering the lower *κ* of SiGe. Unexpectedly, most other samples show significantly lower *κ* compared with SiGe/Si even though the inclusions have a higher bulk *κ*. The effect of nanoinclusions on *κ* is generally attributed to their size and density differences between the nanoinlcusions and matrix^22^. However, it seems that our results cannot be simply explained by these factors. For instance, for the same configuration with *d_S_* of 1.1nm and *d_P_* of 1.1nm, *κ* (6.9W/mK) of Ge110/Si is less than one quarter of that (30.9W/mK) of Ge100/Si although *κ* of Ge is isotropic. Actually, the embedding of nanoinclusions also introduces lattice constant mismatch and interatomic potential mismatch except for density difference. In MD simulations, we can individually investigate the effect of each factor although these factors usually couple together. It is found that the lattice constant mismatch is typically < 1% for the relaxed samples. To evaluate the effect of lattice constant mismatch, we modified the potential of Ge to eliminate this mismatch while keeping other properties the same. The simulations show that *κ* of Ge100/Si with the modified potential is very close to that of the original one (*κ* = 30.9W/mK), indicating a negligible influence of the small lattice constant mismatch. This agrees with the previous observation that *κ* changes little when the strain caused by lattice constant variation is less than 1% in bulk Si^37^. We then investigated the effects of density difference and potential mismatch on *κ* by modifying the atomic mass and potential of the nanoinclusions in the Ge100/Si. In Sample A, the potential for Ge is replaced by that of Si while in Sample B the atomic mass of Ge is changed to that of Si. It is found that *κ* of the former (31.2W/mK) changes little while the latter exhibits a *κ* value close to that of bulk Si. We further changed the atomic mass of nanoinclusions and found that *κ* decreases with enhanced density difference, agreeing with previous investigations^22^. Based on the above analysis, one can conclude that density difference is the main reason that causes the reduced *κ* in Ge100/Si nanocomposites while interatomic potential and small lattice constant mismatches considered here have little effect.

Although density difference accounts for the reduction of *κ* in Ge100/Si, the mechanism that leads to the further reduction of *κ* in Ge110/Si and Ge111/Si is still unclear. Considering the isotropic thermal properties in Ge, there should be little difference for *κ* between them. Therefore, the difference in *κ* is probably due to the lattice orientation mismatch. We note that the samples can be divided into two groups based on the lattice structures of nanoinclusions: lattice-structure-matched nanocomposites, including Ge100/Si and SiGe/Si, and lattice-structure-mismatched ones, i.e., the lattice orientation and/or group symmetry of the nanoinclusions are different from those of the matrix. The significant difference between *κ* of these two groups indicates that these mismatches may be crucial for the further reduction of *κ*.

**Figure 3:**
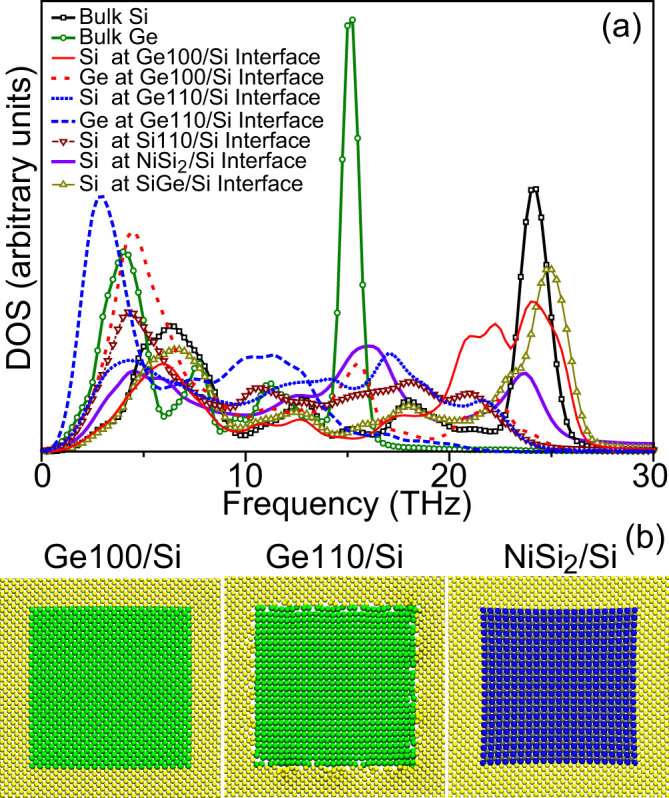
(a) Phonon density of states of Si and Ge atoms at the interfaces in the samples Ge100/Si, Ge110/Si, Si110/Si, SiGe/Si and NiSi_2_/Si with respect to those of bulk. (b) Relaxed structures of Ge100/Si, Ge110/Si and NiSi_2_/Si nanocomposites at 300K.

Due to the mismatches in lattice orientation and/or group symmetry, the vibrational properties of interfacial atoms may be affected. Figure 3(a) shows the PDOS of interfacial Si and Ge atoms in Ge100/Si, SiGe/Si, Ge110/Si, Si110/Si and NiSi_2_/Si, with respect to those of bulk Si and Ge. For all the samples, there are significant PDOS mismatches between the interfacial atoms, accounting for the interfacial scattering and thus the reduced *κ*. For example, the interfacial Si (Ge) atoms in Ge100/Si and SiGe/Si vibrate at higher frequencies than those in the bulk, which has also been reported in the study of other solid-solid interfaces^29^,^38^,^39^. These higher frequency phonons only exist close to the interface and cannot participate in the energy transport across interface without phonon scatterings, because they are significantly mismatched with phonon modes far from the interface. The new observations mainly lie in the lower frequency regime. For Ge100/Si and SiGe/Si, the acoustic PDOS of interfacial Si (Ge) atoms below 5THz almost overlap with those of the bulk although their optical PDOS are significantly suppressed. However, in Ge110/Si, Si110/Si and NiSi_2_/Si, the acoustic PDOS of the interfacial Si and Ge atoms significantly shift to the lower frequency and the optical peaks of PDOS are further reduced with respect to those in Ge100/Si and SiGe/Si. Such a shift is caused by the destruction of lattice periodicity near the interfaces. For the interfaces in Ge100/Si and SiGe/Si, only element disorder arises and lattice periodicity is preserved, which mainly affects optical phonons but has much less influence on acoustic phonons. However, for the interfaces in Ge110/Si, Si110/Si and NiSi_2_/Si, there is also lattice disorder due to the mismatches in lattice orientation or space symmetry except for the element disorder, which therefore remarkably disturbs acoustic phonons and has stronger influence on optical phonons. Because acoustic phonons dominate the thermal transport in Si and Ge, the acoustic PDOS mismatch can strongly suppress the acoustic phonon transport and thus reduces *κ* to the alloy limit. The PDOS mismatch in Si110/Si is as strong as that in Ge110/Si, indicating that the lattice disorder almost dominates the PDOS mismatch. If there is a large difference between the lattice arrangements of interfacial atoms, atomic reconstruction usually happens^38^ and modifies the lattice periodicity of interfacial atoms. As shown in Fig. 3(b), there is a significant atomic reconstruction for the interfacial atoms in Ge110/Si while another two samples almost remain their initial lattice positions. In Ge100/Si and NiSi_2_/Si, the lattice arrangements of nanoinclusions are very similar to that of the matrix, which makes the interfacial atoms only deviate slightly from their initial positions. However, the difference in interfaces between Ge110/Si and NiSi_2_/Si indicates that acoustic PDOS mismatch may also arise even when there is no significant atomic reconstruction. This is because the vibrational properties of an atom are related to its interactions with surrounding atoms and atomic reconstruction is just one possible way to change the coupling.

**Figure 4:**
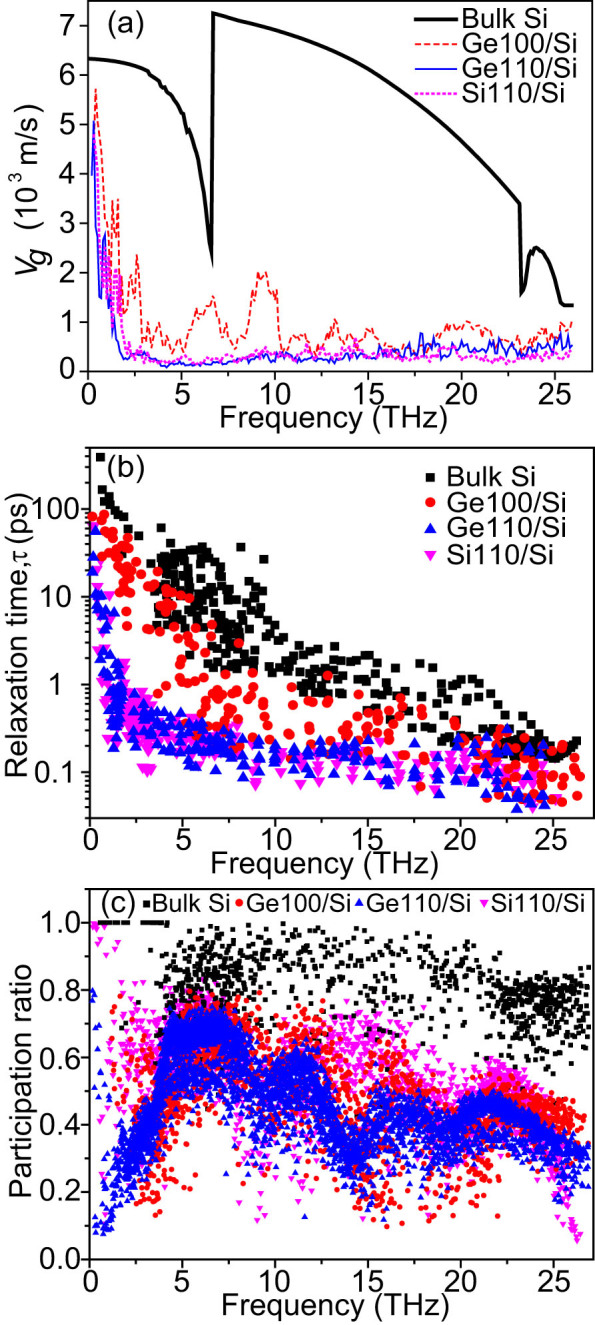
Average phonon group velocities (a), relaxation times (b) and participation ratios (c) for the samples Ge100/Si, Ge110/Si, Si110/Si and bulk Si at 300K.

To clarify the effects of lattice orientation mismatch on the phonon transport in Si-based nanocomposites, we calculated the phonon group velocities *V_g_* and relaxation times *τ* of Ge100/Si, Ge110/Si, and Si110/Si, in comparison with those of bulk Si, as shown in Figs. 4(a) and (b). All the samples used in the phonon spectrum analysis have the same *d_S_* of 1.1nm and *d_P_* of 1.1nm. The group velocities were obtained by numerically differentiating the phonon dispersions extracted from EMD simulations^40^. The relaxation times were obtained by fitting the autocorrelation function of the total energy of each phonon mode with an exponential function^34^,^41^. Obviously, group velocities of Ge100/Si, especially for optical modes, are greatly reduced with respect to those in bulk Si, which can be attributed to the lower group velocities in nanoinclusions and the zone folding effects, as reported in nanoporous structures^34^,^42^. When the frequency approaches 0, group velocities in Ge100/Si are comparable to those in bulk Si but they rapidly decrease with the increasing frequency and remain relatively low values at higher frequencies. The reduction of relaxation times in these nanocomposites is also significant over most frequency range. More importantly, the group velocities and relaxation times of most phonons in Ge110/Si and Si110/Si are further reduced with respect to those of Ge100/Si. This can be ascribed to the enhanced boundary scattering caused by the acoustic PDOS mismatch and enhanced optical PDOS mismatch. One can find much shorter relaxation times in Ge110/Si from 1 to 5THz, agreeing well with the frequency range where acoustic PDOS mismatch happens. Due to the same configuration and the dominance of boundary scattering, Ge110/Si and Si110/Si exhibit similar group velocities and relaxation times.

It has been reported that interfaces/surfaces may result in phonon localizations in Si nanotubes^43^ and Ge-Si core-shell nanowires^44^. To understand the localization effect, the participation ratiosof phonon modes in these samples were calculated, as shown in Fig. 4(c). The participation ratios of most modes in the nanocomposites are significantly reduced with respect to those in bulk Si, indicating that the introduction of nanoinclusions makes phonons more localized. Compared with Ge100/Si, the participation ratios of many modes in Ge110/Si are significantly lower, indicating that lattice-orientation-mismatch leads to the enhancement of localization effect. The localization generally arises at surfaces/interfaces and can be enhanced by disorder^43^. As mentioned above, lattice-orientation-mismatch induces lattice disorder at the interfaces, which therefore enhances the localization. Similar behavior has been observed in nanoporous Si^45^.

Therefore, one can conclude that the further reduction of *κ* in Ge110/Si and Si110/Si compared to Ge100/Si is mainly caused by the lattice orientation mismatch, which results in the significant reduction of group velocities and relaxation times, as well as the enhancement of phonon localizations. Similar phenomena are also found for Ge111/Si and Si111/Si. Compared with Si110/Si and Si111/Si, Ge110/Si and Ge111/Si show slightly lower *κ*, which is likely due to the additional scatterings caused by element mismatch. Obviously, boundary scattering dominates the thermal transport in these nanocomposites, making their *κ* values similar for the same configurations. The dominance of the boundary scattering also explains why *κ* of lattice-structure-mismatched nanocomposites are almost independent on *d_P_*.

**Figure 5:**
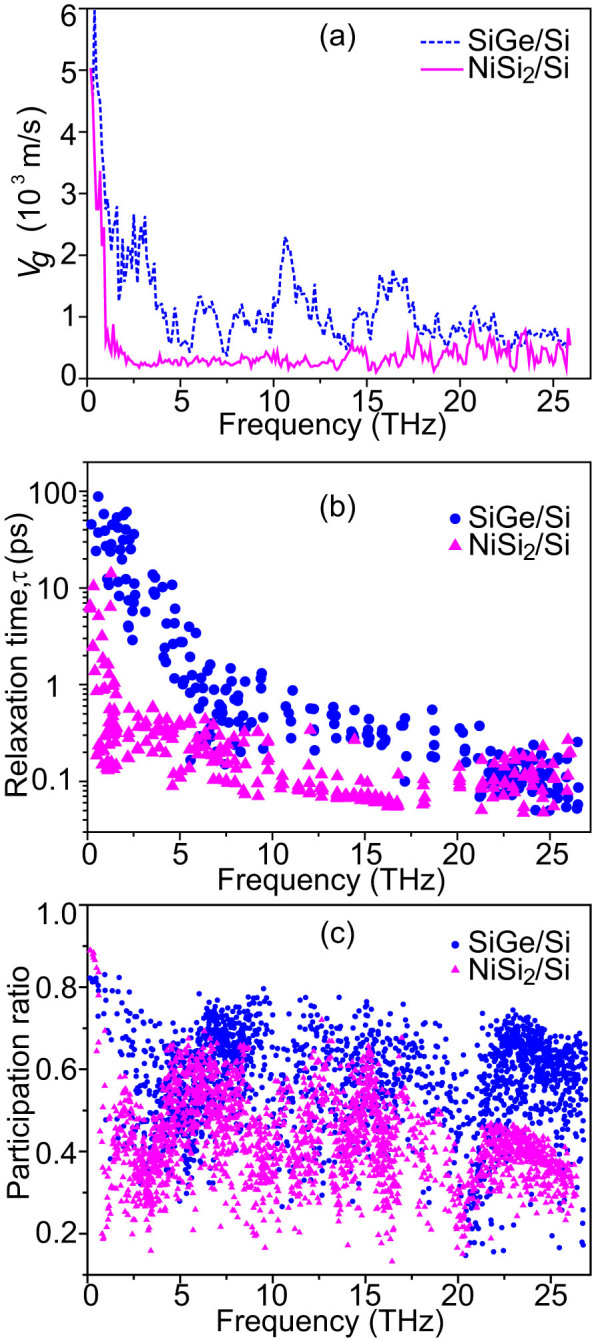
Average phonon group velocities (a), relaxation times (b) and participation ratios (c) for the samples SiGe/Si and NiSi_2_/Si at 300K.

Similarly, the acoustic PDOS mismatch caused by different space symmetries also greatly suppresses the phonon transport in nanocomposites. As shown in Fig. 2, NiSi_2_/Si shows a substantially lower *κ* than that of SiGe/Si whose nanoinclusions have the same space symmetry as the matrix, although *κ* of NiSi_2_ is even higher than that of SiGe. The acoustic PDOS mismatch also significantly depresses group velocities and relaxation times in NiSi_2_/Si, as shown in Figs. 5(a) and (b). Over most frequency range, especially between 1 and 5THz, NiSi_2_/Si nanocomposite exhibits significantly lower group velocities and shorter relaxation times with respect to those in SiGe/Si. The enhancement of phonon localization in NiSi_2_/Si is obvious, as marked by the significantly lower participation ratios of most modes compared with those in SiGe/Si. Thus, space-symmetry-mismatch accounts for these variations and the further reduction of *κ* in NiSi_2_/Si.

The above analysis shows that the lattice orientation and space symmetry mismatches can lead to significant acoustic PDOS mismatch near interfaces and thus further reduce *κ* of nanocomposites to the alloy limit by depressing group velocities and relaxation times, as well as enhancing phonon localizations. For thermoelectric applications, metal silicides may be preferable fillers because of their better electrical properties. Several investigations suggest that nanoinclusions may enhance the power factor with respect to that of the matrix, e.g., the power factor can be improved in InGaAs embedded with ErAs that can act as electron donor^2^,^22^. Such a low *κ* combined with even improved electrical properties may make Si-based nanocomposites a promising candidate for thermoelectric applications. In addition, the lattice structure mismatches can significantly suppress low frequency phonons that are generally difficult to block, providing a new strategy to suppress acoustic phonon transport.

**Figure 6:**
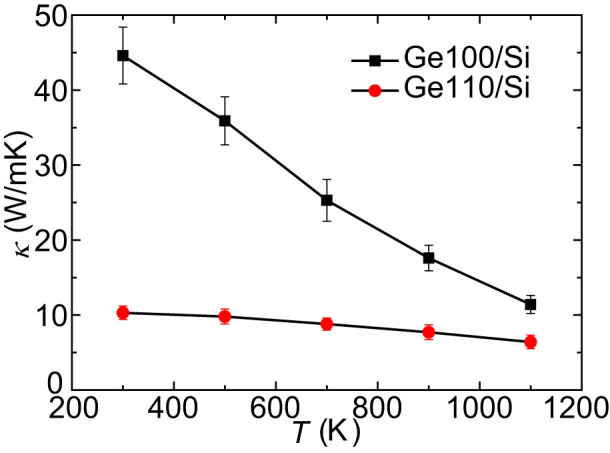
Thermal conductivities of the samples Ge100/Si and Ge110/Si as a function of temperature.

Next, we investigated the effects of lattice structure mismatch on the temperature dependence of *κ*. Figure 6 shows *κ* of Ge100/Si and Ge110/Si with *d_S_* of 2.2nm and *d_P_* of 1.1nm between 300K and 1100K. Significantly different temperature dependences are observed. *κ* of Ge110/Si is insensitive to temperature while that of Ge100/Si notably decreases as the temperature rises. It is found that the MFPs of most modes in Ge110/Si are less than or comparable with the interatomic distance and those phonons with MFPs significantly larger than the interatomic distance only contribute ~ 6% to *κ*. The dominant modes with short MFPs mainly limited by the strong boundary scattering are hardly affected by temperature. Therefore, the application temperature range for these nanocomposites will be significantly broadened. Similar temperature dependence of *κ* was also observed in nanoporous SiGe alloy^46^.

**Figure 7:**
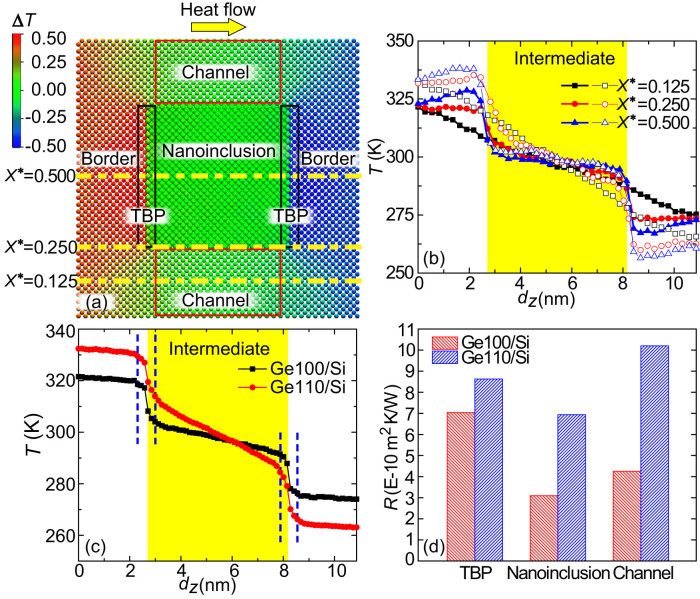
(a) Temperature distribution in the Ge100/Si nanocomposite. (b) Temperature variations at the lines X* = 0.125, 0.250 and 0.500 (the solid symbols represent the results for Ge100/Si and the empty symbols indicate those for Ge110/Si). (c) The average temperature profiles along the *Z* direction in the samples Ge100/Si and Ge110/Si by applying the same heat flux. (d) Thermal resistances of TBP, nanoinclusion and channel regions for the samples Ge100/Si and Ge110/Si.

The spectrum analysis provides an overall frequency-domain explanation of the effect of lattice orientation and space symmetry mismatches on the thermal transport. Understanding the temperature distribution and thermal resistance related to the configurations will provide more details about local thermal transport in the real space and help with the design and optimization of nanocomposites. We therefore calculated the temperature distributions and profiles for the Ge100/Si and Ge110/Si with *d_S_* = 5.4nm and *d_P_* = 5.6nm by applying the same heat flux. The temperature of a group of *N* atoms is defined by their time-averaged kinetic energy *E_k_*, *T* = 2 <*E_k_*>/(3*Nk*_B_) (*k*_B_ is the Boltzmann constant), indicating the local energy density. Figure 7(a) shows the temperature distribution of Ge100/Si for the heat flux along the *Z* direction, indicating an obvious nonuniformity. Figure 7(b) further shows the temperature variations along the normalized coordinates X* = 0.125, 0.250 and 0.500 in Ge100/Si and Ge110/Si. The temperature distributions and profiles show that a temperature jump arises at the interfacial regions. For the line away from the nanoinclusion (X* = 0.125), temperature decreases monotonically. But for the lines passing through the interface (X* = 0.250) and nanoinclusion center (X* = 0.500), abnormal temperature gradients are observed. The temperature slope for the line X* = 0.500 is negative in the Border regime, showing a clear hot region in front of the nanoinclusions and a cold region behind them, which are caused by the phonon energy accumulation and depletion, respectively. This phenomenon probably results from phonon ballistic transport through channels, as explained by Yang et al.^18^. Similar behavior has also been observed in nanoporous structures^34^. The overall temperature profiles along the heat flow direction are shown in Fig. 7(c). It is clear that the total temperature drop for Ge110/Si is significantly larger than that in Ge100/Si, corresponding to a lower *κ* in the former. The temperature slope of Ge110/Si in the intermediate region is more than two times larger than that of Ge100/Si, indicating increased thermal resistance due to the lattice orientation mismatch.

Because the heat flux distribution is also nonuniform in nanocomposites, thermal resistances of the thermal boundary phase (TBP), nanoinclusions between TBP and channel regions were then calculated to quantitatively evaluate the influence of lattice orientation mismatch, as shown in Fig. 7(d). The TBP consists of half UC on the matrix side and half UC on the nanoinclusion side because the PDOS of atoms within half UC near the interfaces are significantly modified. From the values of temperature difference Δ*T*, heat flow *J* and cross section area *A_c_*, the thermal resistance *R* can be determined by *R* = Δ*TA_c_*/*J*. The thermal resistance of the Border region is a summation of those in the left and right Borders. The TBP thermal resistance *R*_TBP_ includes the contributions from the two TBPs. Clearly, lattice orientation mismatch strongly increases the thermal resistance of the nanoinclusion and channel regions while only slightly enhances *R*_TBP_. In the nanoinclusion and channel regions, the enhanced boundary scattering due to the PDOS mismatch significantly decreases the MFP and thus results in increased thermal resistances. Similarly, it has been reported that the strong boundary scattering determines the thermal resistance of channel regions that dominate the thermal transport in nanoporous Si^34^. In both samples, *R*_TBP_ is important while *R*_TBP_ of Ge110/Si is only 23% larger than that of Ge100/Si. The variation of *R*_TBP_ in both samples can be rationalized by the diffuse mismatch model (DMM)^47^, which provides relatively good agreements with experiments at room or higher temperatures^38^,^48^. When the PDOS near the interface are used, DMM predicts a 20% larger *R*_TBP_ for Ge110/Si in comparison with that in Ge100/Si, agreeing well with the NEMD simulation results. Similarly, the thermal resistances of different regions in NiSi_2_/Si are enhanced due to the space symmetry mismatch.

## Discussion

In summary, we have calculated *κ* of single-crystalline Si-based nanocomposites embedded with various nanoinclusions using NEMD simulations and conducted a phonon transport analysis in both frequency domain and real space domain. Specifically, we designed nanoinclusions with a lattice orientation or space symmetry different from that of the matrix. It was found that *κ* of nanocomposites with lattice-structure-mismatched nanoinclusions are significantly lower than those of the lattice-structure-matched ones and can even approach the alloy limit. A comparison between the PDOS of interfacial atoms and bulk shows a significant acoustic PDOS mismatch and enhanced optical PDOS suppression due to the destruction of lattice periodicity in nanocomposites, which therefore introduce strong scatterings for acoustic and optical phonons respectively. Further phonon spectrum analysis shows that the nanocomposites with lattice-structure-mismatched nanoinclusions exhibit notably lower group velocities, shorter relaxation times and stronger phonon localizations. Specifically, the low frequency phonons are greatly suppressed due to the destruction of lattice periodicity, providing a new strategy to block long-MFP phonons.

Additionally, the lattice structure mismatch leads to a temperature-insensitive *κ* in Si-based nanocomposites from 300K to 1100K, which can be ascribed to the very short MFPs of dominant phonon modes caused by strong boundary scattering. The local thermal transport details were further illuminated by exploring the temperature distributions and profiles in the nanocomposites, showing that with the introduction of lattice structure mismatch, the thermal resistance of interfacial regions only slightly increases while those of nanoinclusions and channels between them are remarkably improved. This is due to the enhanced boundary scattering at the interfaces parallel to the heat flux.

Approaching the alloy limit of *κ* combined with even improved electrical properties will significantly improve the *ZT* of single-crystalline Si-based nanocomposites, which thus may become a promising bulk thermoelectric candidate. Especially, the insensitive temperature dependence of *κ* in these nanocomposites will therefore extend their applications to a wide temperature range.

## Method

### NEMD simulation

The NEMD simulations were performed in LAMMPS^49^ using the direct method^50^, with the periodic boundary condition applied to the *X*, *Y* directions and the fixed boundary condition to the *Z* direction. The MEAM potential parameters for Si and Ge are from Refs. 26 and 27, respectively. The cross potential parameters for Si-Ge and Ni-Si are from Refs. 24 and 25, respectively. The nanoinclusions are embedded in the intermediate region of the simulation domain, which is separated with the heat source/sink by a buffer layer to eliminate the nonlinear effect. Initially, the system was equilibrated at a given temperature within the *NPT* (constant number of particles *N*, pressure *P* and temperature *T*) ensemble with a Nose-Hoover thermostat for 300ps and then in the *NVE* (constant number of particles *N*, volume *V* and energy *E*) ensemble for 100ps. The two ends of the simulation domain, with a thickness of one unit cell (UC), were in contact with Langevin thermostats to form the heat source and sink. The temperature of the heat source and sink was set as *T*_H_ = *T*_0_ + Δ/2 and *T*_C_ = *T*_0_ Δ/2, respectively, where *T*_0_ is the mean temperature of the system and Δ is the temperature difference. After the system reached a steady state, a linear temperature profile was achieved in the intermediate region for 36ns. The time-averaged temperature profile in the intermediate region was linearly fitted to obtain the temperature gradient, which was used to calculate the thermal conductivity according to Fourier's law
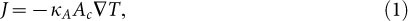
where *J* is the heat flow and *A_c_* is the cross section area. To eliminate the finite-size effects, for each configuration, a series of simulations with variable domain lengths (*L_Z_*) ranging from 65.2nm to 273.7nm were performed. The thermal conductivity in bulk limit *κ* was obtained by fitting to 1/*κ_A_* - 1/*L_Z_* relation under the gray approximation and extrapolating to *L_Z_* → ∞^50^, where a good linear correlation was found.

### Participation ratio

Phonon localization can be quantitatively characterized by the participation ratio *p_λ_* defined for each eigenmode *λ* as^51^
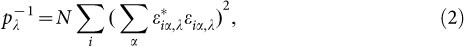
where *N* is the total number of atoms and *ε*_iα,λ_ is the *α*th eigenvector component of eigenmode *λ* for the *i*th atom. The participation ratio measures the fraction of atoms participating in a given mode and effectively indicates the localized modes with *O*(1/*N*) and delocalized modes with *O*(1).

### Diffuse mismatch model

According to the diffuse mismatch model, the phonon boundary resistance *R_b_* between two materials 1 and 2 is given by
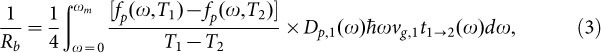
where *ω_m_* is the cutoff frequency of the softer material, 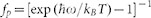 is the BoseEinstein (equilibrium) distribution, *D_p_* is the PDOS of interfacial atoms and *v_g_* is the phonon group velocity. *t*_1 → 2_ is the transmission coefficient from the material 1 to 2 and can be calculated based on the phonon mode *λ* by
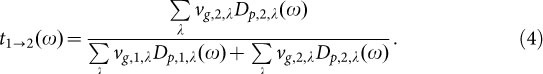


## Author Contributions

R.G. carried out the calculations and data analysis and prepared all the figures. R.G. and B.H. wrote the manuscript. All authors have reviewed, discussed and approved the results and conclusions of this article.
